# Cellular and Molecular Insights Into the Etiology of Subfertility/Infertility in Crossbred Bulls (*Bos taurus* × *Bos indicus*): A Review

**DOI:** 10.3389/fcell.2021.696637

**Published:** 2021-07-08

**Authors:** Arumugam Kumaresan, Kamaraj Elango, Tirtha Kumar Datta, Jane M. Morrell

**Affiliations:** ^1^Theriogenology Laboratory, Southern Regional Station of Indian Council of Agricultural Research (ICAR)-National Dairy Research Institute, Bengaluru, India; ^2^Animal Genomics Laboratory, Indian Council of Agricultural Research (ICAR)-National Dairy Research Institute, Karnal, India; ^3^Swedish University of Agricultural Sciences, Uppsala, Sweden

**Keywords:** crossbred bulls, testis, sperm molecules, infertility, sub-fertility

## Abstract

Crossbreeding of indigenous cattle (*Bos indicus*) with improved (*Bos taurus*) breeds gained momentum and economic relevance in several countries to increase milk production. While production performance of the crossbred offspring is high due to hybrid vigor, they suffer from a high incidence of reproductive problems. Specifically, the crossbred males suffer from serious forms of subfertility/infertility, which can have a significant effect because semen from a single male is used to breed several thousand females. During the last two decades, attempts have been made to understand the probable reasons for infertility in crossbred bulls. Published evidence indicates that testicular cytology indices, hormonal concentrations, sperm phenotypic characteristics and seminal plasma composition were altered in crossbred compared to purebred males. A few recent studies compared crossbred bull semen with purebred bull semen using genomics, transcriptomics, proteomics and metabolomics; molecules potentially associated with subfertility/infertility in crossbred bulls were identified. Nevertheless, the precise reason behind the poor quality of semen and high incidence of sub-fertility/infertility in crossbred bulls are not yet well defined. To identify the underlying etiology for infertility in crossbred bulls, a thorough understanding of the magnitude of the problem and an overview of the prior art is needed; however, such systematically reviewed information is not available. Therefore, the primary focus of this review is to compile and analyze earlier findings on crossbred bull fertility/infertility. In addition, the differences between purebred and crossbred males in terms of testicular composition, sperm phenotypic characteristics, molecular composition, environmental influence and other details are described; future prospects for research on crossbred males are also outlined.

## Introduction

Approximately 150 million rural households (750 million people) around the world are engaged in milk production, mainly in developing countries (FAO et al., 2018). The dairy sector could provide real hope for a sustainable income in rural households in many tropical and sub-tropical countries. Therefore, strategies to reduce global hungry and poverty are proceeding through dairy development (FAO et al., 2018, 2020). To improve milk production, crossbreeding between *Bos taurus* and *Bos indicus* is being practiced in several countries (Singh, 2016; Khan et al., 2018) resulting in offspring with a blend of desirable characters, such as high milk yield, long lactation length, early maturing ability, earlier age at first calving and shorter calving interval (Galukande et al., 2013; Leroy et al., 2016; Ema et al., 2018). Crossbred animals improved the livelihood of impoverished farmers and metamorphosed several tropical/sub-tropical countries from deficient to sufficient/efficient in terms of milk production. In contrast, compromised reproductive performance of crossbred animals is a major constraint faced by the farmers in these countries. Studies conducted on crossbreeding schemes in tropical regions revealed that milk production showed higher (35.13%) heterosis but fertility showed only moderate (12.02%) heterosis (Bunning et al., 2019). Since the focus was on milk production, reproduction was frequently overlooked, with the result that infertility problems in crossbred cattle persist. Thus, for instance, reproductive problems are higher in crossbred (43.7%) than indigenous (24.5%) cows in south west Ethiopia (Molalegne and Shiv, 2011). Even though infertility is common in both crossbred males and females, infertility in a male can have a formidable effect since semen from a single bull is used for artificial breeding of thousands of cows (Kastelic, 2013). Female fertility received much attention and was enhanced through assisted reproductive technologies, as well as genetic selection, whereas bull fertility was largely ignored (Butler et al., 2020). Globally, a significant proportion of reproductive failure is attributable to bull subfertility due to poor semen quality (DeJarnette et al., 2004).

Infertility in a bull is defined as the inability to achieve pregnancies, whereas sub-fertile bulls (i.e., with reduced fertility) delay conception, prolong the calving season, reduce calf weaning weights, and increase the numbers of females culled, thereby resulting in economic losses and threatening the sustainability of a livestock operation (Kastelic, 2013). Breed variations in the incidence of infertility are well-documented. Among different breeds, poor semen quality and sub-fertility/infertility are the major reasons for culling of taurine × indicine crossbred bulls, despite being the progeny of best dams and confirmed sires (Mukhopadhyay et al., 2010; Khatun et al., 2013; Thippeswamy et al., 2014). Several authors reported that almost 80–90% of purebred animals have no reproductive problems (Kennedy et al., 2002; Bjelland et al., 2011). In contrast, a greater proportion of bulls with reduced potential fertility was found in hybrids in comparison with their parental pure breeds (Horn et al., 2005). A synthetic breed (i.e., composite or hybrid or stabilizer) is a new breed or line from crossing two or more existing breeds, especially to increase hybrid vigor (Chacko, 2005). Chacko (2005) observed that only 27% of synthetic bulls produced good quality semen. Similarly, out of 414 Holstein Friesian crossbred bulls, only 25.64% bulls produced quality semen that could be successfully cryopreserved for use in artificial breeding (Khatun et al., 2013). Rearing crossbred calves or bulls is very expensive as they require better nutrition and more stringent disease-control strategies than indigenous bulls; therefore, culling adult bulls due to sub-fertility/infertility leads to huge economic loss.

The male offspring born out of species hybridization (for instance, crossing cattle with yak) are always sterile. The male progeny born via crossbreeding of a *Bos taurus* male with *Bos indicus* female, although not sterile, show increased incidences of sub-fertility and/or infertility compared to their parents (Thippeswamy et al., 2014). Despite their common ancestral base, Asiatic zebu cattle (*Bos indicus*) and European taurine cattle (*Bos taurus*) exhibit several morphological and physiological differences. At chromosome level, the Y-chromosome in *Bos taurus* is submetacentric while that in *Bos indicus* is acrocentric. Therefore, it was proposed that the lower fertility in zebu crosses with European cattle could be due to small deletions or position changes between the synapse region of the X and Y chromosomes, or to alterations in genes participating in the regulation of reproduction (Horn et al., 2005). With the advancements in science and analytical techniques, nowadays, there is an increased interest in identification of male fertility markers. Several studies used genomics, transcriptomics, proteomics and metabolomics approach to ascertain molecular determinants of bull fertility. Few studies used high throughput techniques for assessment of transcriptomic, proteomic and metabolomic differences between semen of high- and low-fertile bulls and identified potential molecules for fertility prediction (Peddinti et al., 2008; D’Amours et al., 2010; Card et al., 2013, 2017; Prakash et al., 2021). While these approaches offer a great scope for prediction of bull fertility, reported variations in sperm molecules among different breeds (Aslam et al., 2015) indicate that fertility associated semen molecules might vary with breed, which in turn demand identification of breed specific fertility markers.

Despite these fertility problems, crossbreeding is commonly adopted to improve the milk productivity of indicine and non-descript cattle; the reason for the higher incidence of sub-fertility or poor semen quality in crossbred bulls compared to purebred bulls is not fully understood. Understanding the reason behind infertility/subfertility in crossbred bull will assist development of strategies to improve crossbred bull reproduction. Therefore, in this review, we compiled and analyzed earlier findings on crossbred bull fertility/infertility. In addition, the differences between purebred and crossbred males in terms of testicular composition, sperm phenotypic characteristics, molecular composition, environmental influence, and other details are described and future prospects for research on crossbred males are also outlined.

## Magnitude of Reproductive Problems in Crossbred Males

### High Incidence of Poor Semen Quality

**FIGURE 1:**
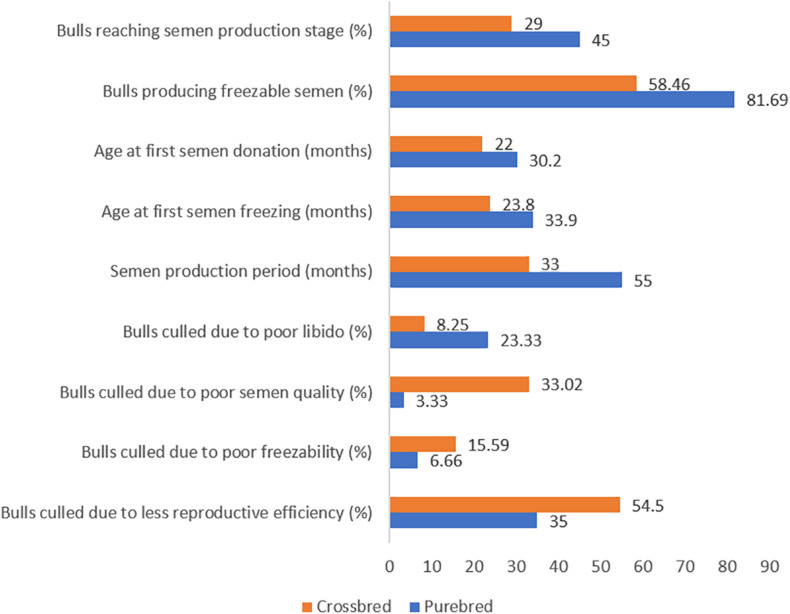
Culling pattern and semen production characteristics in purebred and crossbred bulls (based on the data from Suryaprakasam and Rao, 1993; Rao and Rao, 1995; Rao et al., 1995; Khate, 2005; Mukhopadhyay et al., 2010).

Even though semen volume is reported to be higher in crossbred bulls, seminal quality parameters such as mass activity (Mukhopadhyay et al., 2010), concentration (Sarder, 2003), and total motile sperm count (Isnaini et al., 2019) are higher in indigenous bulls. The proportion of live spermatozoa was higher (70.4–92.2%) in purebred bulls (indigenous and exotic) compared to crossbred bulls (64.8–75.4%) (Singh et al., 2013). The proportion of sperm head abnormalities was significantly higher in *B. indicus* × *B. taurus* (22%) bulls than purebred *Bos indicus* (13%) and *Bos taurus* (15%) bulls (Chacón et al., 1999). More than half (54.96%) of crossbred bulls did not produce ejaculates that meet the minimum standards required for semen freezing (Mathur et al., 2002). Among the bulls who produced non-freezable quality semen, 66.67% were astheno-normozoospermic, 28.70% were oligo-asthenozoospermic and the remainder (4.63%) were normozoo-spermic (Mandal et al., 2012). Ejaculate rejection rate in crossbred bulls ranged from 10 to 100% with the average being 52–55% (Sudheer and Xavier, 2000; Tyagi et al., 2000; Mukhopadhyay et al., 2010; Vijetha et al., 2014; Gopinathan et al., 2016). Ejaculates were rejected for one or more reasons, including low sperm concentration, poor sperm motility and viability. Comparative semen production details of crossbred bulls and purebred bulls are given in Figure 1.

**FIGURE 2:**
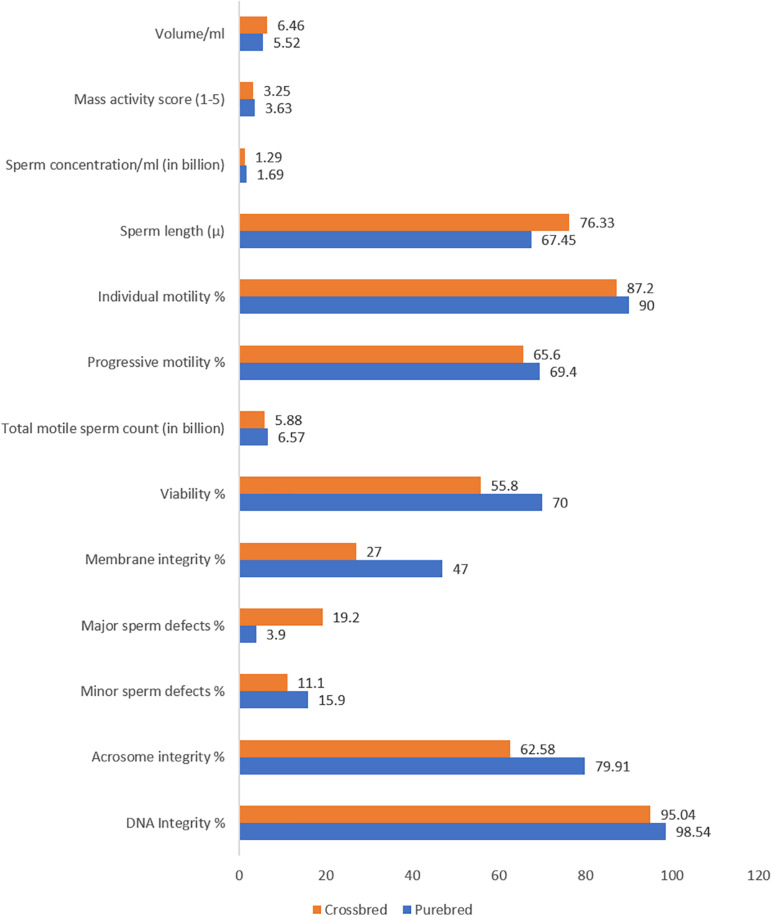
Semenological differences between purebred and crossbred bulls (based on data from Singh et al., 2000; Sarder, 2003; Brito et al., 2004; Mukhopadhyay et al., 2010; Zubair et al., 2013; Kumar et al., 2015; Khan et al., 2018; Isnaini et al., 2019; Chung et al., 2019).

Ejaculate quality in crossbred bulls is also influenced by the level of exotic inheritance (Patel et al., 1989), although reports are contradictory. A few researchers reported better semen quality and quantity in crossbred bulls with higher levels of exotic blood (Mathew et al., 1982); however, others reported that post-thaw motility decreased as the exotic inheritance of the crossbred bulls increased (Sagdeo et al., 1990). Crossbred bulls with a higher level of exotic component (Jersey, Holstein Friesian, or Brown Swiss) produced ejaculates with poor sperm cryotolerance (Sagdeo et al., 1991). Bulls with more than 62.5% exotic inheritance and more than one indigenous breed component produced a greater number of ejaculates with poor sperm cryotolerance. For instance, among the bulls with two, three, and four breed combinations, only 46, 36.11, and 34.48% of bulls produced ejaculates of freezable quality (Tyagi et al., 2006). Holstein Frisian crosses had inferior semen quality and freezability compared to Jersey crossbred bulls (Sagdeo et al., 1990). Crossbred bulls with triple inheritance had a higher proportion of total sperm abnormalities than bulls with double inheritance (Gupta et al., 1990; Prasad et al., 1990; Singh and Pangawkar, 1990). Sperm tail abnormalities were significantly higher in triple crosses than in double crosses (Pande et al., 1994). Nearly 28.3% of the Frieswal bulls showed abnormal detached heads in their spermiogram (Pant et al., 2002). The acrosome of crossbred bull (Triple cross) spermatozoa was more fragile and prone to damage than in exotic purebred bulls. Specific acrosome defects, such as ruffled, knobbed, denuded, swollen, and incomplete acrosome patterns, were reported more often in spermatozoa from crossbred bulls than from exotic purebreds (Sharma et al., 1990). The differences in semen characteristics between crossbred and purebred bulls are shown in Figure 2.

### High Incidence of Sub-Fertility and Culling Rates

The incidence of sub-fertility/infertility in crossbred bulls is high compared to purebred bulls. Sire conception rate was higher in indigenous bulls (51.1%, based on 15558 AI), compared to Holstein Friesian crossbred (47.1%, based on 47396 AI) and Jersey crossbred (43.5%, based on 2751 AI) bulls (Potdar et al., 2020). Another study also reported lower conception rates in crossbred bulls (41.36% in HF crossbred and 39.84% in Jersey crossbred) compared with indigenous (54.26% in Dangi and 45.23% in Khillar) and exotic (44.05% in HF and 42.22% in Jersey) purebred bulls (Potdar et al., 2016). Bhagat and Gokhale (2016) also reported lower conception rates in crossbred bulls (Jersey cross 54.80%, and HF crossbred 52.94%) than indigenous bulls (Dangi-67.21%, Khillar-64.81%, Gir-63.87%, and Sahiwal-61.48%). The cryotolerance level of crossbred bull spermatozoa is inferior to purebred bull spermatozoa; the characteristics of equilibrated and cryopreserved spermatozoa in purebred and indigenous bulls are given in Table 1. The process of equilibration (maintaining extended spermatozoa at 4°C for 4 h) did not affect sperm quality significantly, with the initial values of sperm motility and viability being largely unaltered after equilibration. However, after freezing and thawing, the phenotypic characteristics and functionality of crossbred bull spermatozoa were significantly altered compared to purebred bulls, which might be a reason for high incidence of sub-fertility in these bulls.

**TABLE 1:** Cryo-tolerance between purebred and indigenous bulls (based on the data from Kumar et al., 2015; Khan et al., 2018; Chung et al., 2019; Isnaini et al., 2019).

Seminal parameters	Purebred bulls	Crossbred bulls
**Post-chilled sperm characteristics**		
Individual motility %	89.60 ± 0.86 (Jersey)	88.40 ± 1.96 (Sahiwal × Frieisan)
	56.1 ± 0.22 (Simmental)	55.7 ± 0.21 (Ongole crossbred)
Progressive motility %	69.40 ± 3.61 (Jersey)	67.00 ± 4.44 (Sahiwal × Frieisan)
Viability %	71.72 ± 1.09 (Jersey)	67.91 ± 1.20 (Jersey cross)
**‘Post-thaw sperm characteristics**		
Individual motility %	76.54 ± 0.55 (Sahiwal)	40.00 ± 0.35 (HF × Sahiwal)
	52.75 ± 13.08 (Jersey)	37.00 ± 13.45 (HF × Sahiwal)
	41.1 ± 0.22 (Simmental)	40.9 ± 0.21 (Ongole crossbred)
Progressive motility %	37.5 ± 10.00 (Jersey)	20.40 ± 8.86 (HF × Sahiwal)
Dead sperm %	19.04 ± 0.50 (Sahiwal)	51.87 ± 0.50 (HF × Sahiwal)
Viability %	58.67 ± 1.02 (Jersey)	51.63 ± 0.97 (Jersey cross)
Membrane Integrity %	78.58 ± 0.45 (Sahiwal)	38.29 ± 0.45 (HF × Sahiwal)
Acrosome integrity %	71.94 ± 0.86 (Jersey)	69.38 ± 0.53 (Jersey cross)
	70.95 ± 0.47 (Sahiwal)	35.83 ± 0.56 (HF × Sahiwal)
DNA Integrity %	96.70 ± 0.16 (Sahiwal)	92.45 ± 0.28 (HF × Sahiwal)

Higher culling rates (40–70%) due to poor semen quality, poor sperm cryotolerance and sub-fertility/infertility were reported in crossbred bulls (Sethi et al., 1989; Khate, 2005; Khatun et al., 2013). In a study by Khate (2005), 33 and 16% of Holstein Friesian crossbred bulls were culled due to poor semen quality and freezability, respectively. The proportion of ejaculates rejected for artificial breeding due to inferior quality was higher in Holstein Friesian X Sahiwal crossbred bulls (33.7–50.1%) than in Holstein bulls (11.5–33%) (Usmani et al., 1993). The reproductive wastage was twice the level in crossbred bulls compared to purebred bulls for the following crosses: Brown Swiss (28.3%) vs. Crossbred Sunandhini (73.8%) (Mathew et al., 1982), Ongole (16.6%) vs. Jersey × Ongole (38.1%) (Rao et al., 1995), Sahiwal (3.48%) vs. Holstein (19%) vs. Holstein Friesian crossbred (43%) (Patel et al., 2001), and Hereford (18%) vs. Crossbred (47%) (Moraes et al., 1998). In addition, Mukhopadhyay et al. (2010) reported a higher incidence (31.41%) of poor-quality semen in Holstein Friesian crossbred (Karan Fries) bulls. Poor semen quality was the major reason for culling these males Chauhan (2007). In the case of Sunandhini bulls (a composite breed of cattle developed by crossing non-descript cattle with Brown Swiss, Jersey cattle and Holstein Friesian), only 27% of bulls produced good quality semen (Chacko, 2005). In contrast, semen quality was not a problem in indigenous bulls (Mathew et al., 1982; Khate, 2005).

### High Seasonal Variations in Semen Quality

Crossbred bulls tend to have a higher susceptibility to environmental conditions than purebred bulls. The effect of season and breed × season interaction on proportion of freezable quality ejaculates were more pronounced in crossbred cattle than purebred bulls (Sagdeo et al., 1991). In tropical Ethiopia, a seasonal influence on seminal parameters in crossbred (Boran × Holstein) and Boran bulls was found (Tegegne et al., 1994), with inferior semen characteristics being observed in crossbred bulls; there was a significant effect on sperm motility and concentration in the humid season. In Brazil, semen quality of crossbred bulls decreased significantly during hot summer months compared to *Bos indicus* males (Silva et al., 1991). A high environmental temperature was associated with an increase in the number of bulls classified as unfit for breeding. More crossbred bulls (46%) were classified as unfit for breeding than *Bos taurus* (40%) and *Bos indicus* (29%) (Chacón et al., 1999). A significant impact of season was reported on sperm concentration and mass motility in HF × Hariana bulls by Tomar et al. (1985). The lowest values of volume, mass activity, motility and viability in HF × Hariana and Jersey × Hariana bulls were reported during hot-dry season (Goswami et al., 2000). Singh et al. (2000) observed a significant impact of hot and humid climate conditions on semen quality, reaction time and sex drive. Likewise, Bhakat et al. (2014) reported that seminal parameters of Holstein Friesian crossbred bulls were optimal during winter, but intermediate and poor during the rainy and summer seasons, respectively. In contrast, no effect of season on semen quality in crossbred bulls was observed in some studies. Chauhan et al. (2010) reported that season did not have any effect on semen production in crossbred bulls. However, Dhami et al. (1998) reported that excellent quality ejaculates were produced during summer months and poor-quality ejaculates were produced in winter months in Holstein Friesian crossbred bulls. Narasimha Rao et al. (1996) noted higher levels of head, midpiece and tail abnormalities during rainy, winter and summer seasons, respectively. In addition to semen quality, the expression of microRNA in the blood of crossbred bulls was reported to be altered during thermal stress compared to winter season. Among the 420 microRNAs, 65 were dysregulated during peak summer temperatures. The majority of these microRNAs had the Heat shock proteins family genes as their target (Sengar et al., 2018).

## Compounding Factors for Infertility/Sub-Fertility in Crossbred Bulls

In an effort to understand the reasons behind the higher incidence of infertility/sub-fertility in crossbred males, several researchers studied the physiological and molecular differences between crossbred and purebred males. Some studies proposed axiomatic evidences at genetic (Sagdeo et al., 1990), hormonal (Gulia et al., 2010), semenological (Mukhopadhyay et al., 2010), and andrological (Tripathi et al., 2015) levels. However, the precise reasons for higher incidence of infertility/sub-fertility in crossbred males remains elusive. In the following sections, information on the differences at testicular histological, endocrinological and molecular levels between crossbred males and purebred males are compiled and analyzed.

### Alterations in Molecular Composition of Spermatozoa

During spermiogenesis, chromatin compaction occurs by replacing histones with protamines; little or no cytoplasm remains in spermatozoa (Jodar and Oliva, 2014). Therefore, it was previously thought that spermatozoa serve only to deliver paternal DNA to the oocyte. Later, the discovery of RNA in spermatozoa suggested additional roles beyond that of delivering paternal DNA (Alves et al., 2020). Although differences in opinion exist about the transcription and translation activities in spermatozoa, it was observed that paternal RNAs reside in the perinuclear theca of spermatozoa and are transferred during fertilization (Ostermeier et al., 2005). Recent studies profiled sperm RNA and the possible roles in sperm functions and fertilizing ability (Selvaraju et al., 2017; Paul et al., 2020; Prakash et al., 2020). Increasing evidence indicates that expression of sperm molecules, including mRNAs (Wang et al., 2019; Saraf et al., 2021; Selvaraju et al., 2021), proteins (Peddinti et al., 2008; Aslam et al., 2015), phosphoproteins (Kumaresan et al., 2012), and metabolites (Saraf et al., 2020), were altered in bulls with different fertility ratings. All these molecules reflect sperm health and correlate with their fertilizing ability. Breed variations in expression of these molecules are discussed below.

When two different species or breeds are crossed to produce hybrid offspring, the compatibility between spermatozoon and oocyte is altered (Jodar et al., 2013); this compatibility is essential for the transfer of RNA-based information to a chromatized state (Consolidation process). Global transcriptomic profiling of crossbred spermatozoa by next generation RNA Sequencing revealed the transcripts for 13,814 genes, which are highly related to ribosome, spliceosome and oxidative phosphorylation pathways (Prakash et al., 2020, 2021), whereas microarray analysis revealed the expression of 19,454 genes in Vrindavani crossbred bull sperm (Yathish et al., 2017). The *PRM1* is an abundant transcript found in spermatozoa of crossbred bulls (Singh et al., 2019; Prakash et al., 2020), which is also abundant in *Bos indicus* (Raval et al., 2019) and *Bos taurus* (Card et al., 2013; Selvaraju et al., 2017) bulls. However, *PRM1* mRNA expression levels were significantly higher in good quality Holstein Friesian crossbred spermatozoa (Ganguly et al., 2013). Similarly, the circadian rhythm-related CLOCK gene (Bovine circadian locomotor output cycles kaput) and the apoptosis-related CLU (Clusterin) gene were significantly more abundant in good and poor-quality crossbred sperm, respectively (Kumar et al., 2015). Recently, a total of 15,814 and 17,324 transcripts were identified in dwarf zebu bull spermatozoa and crossbred bull spermatozoa respectively, of which 521 transcripts were differentially expressed between purebred and crossbred bull spermatozoa. Furthermore, expression of transcripts involved in ribosome pathway and oxidative phosphorylation were significantly upregulated in crossbred bull spermatozoa compared to purebred bull spermatozoa (DasGupta, 2020). The important sperm transcripts altered in crossbred bull as compared to indigenous bull spermatozoa are shown in Table 2.

**TABLE 2:** Important sperm transcripts downregulated in crossbred bull spermatozoa as compared to indigenous bull spermatozoa (from DasGupta, 2020).

Gene name	Fold change	Functional significance
ENSBTAG00000040064	5.84	Olfactory transduction—G-protein coupled receptor signaling pathway; detection of chemical stimulus involved in sensory perception
MAX (MYC associated factor X)	5.46	MAPK signaling pathway
ENSBTAG00000046639	5.35	Olfactory transduction—G-protein coupled receptor signaling pathway; detection of chemical stimulus involved in sensory perception
ENSBTAG00000030677	4.54	Olfactory transduction—G-protein coupled receptor signaling pathway; detection of chemical stimulus involved in sensory perception
ENSBTAG00000046945	4.30	Olfactory transduction—G-protein coupled receptor signaling pathway; detection of chemical stimulus involved in sensory perception
MRAS (Muscle RAS oncogene homolog)	4.14	Olfactory transduction—signal transduction
ENSBTAG00000026065	4.03	Olfactory transduction—G-protein coupled receptor signaling pathway; detection of chemical stimulus involved in sensory perception
ENSBTAG00000046115		Olfactory transduction—G-protein coupled receptor signaling pathway; detection of chemical stimulus involved in sensory perception
ENSBTAG00000048018	3.59	Olfactory transduction—G-protein coupled receptor signaling pathway; detection of chemical stimulus involved in sensory perception
OR2AT4 (Olfactory receptor family 2 subfamily AT member 4)	3.58	G-protein coupled receptor signaling pathway; detection of chemical stimulus involved in sensory perception

In the case of sperm proteins, four proteins were under-expressed and four proteins were over-expressed in spermatozoa of crossbred bulls compared to purebred bulls (Aslam et al., 2015). Myosin, which is the essential structural component of the sperm and testicular cell, is under- expressed in spermatozoa from crossbred bulls. In addition, beta defensin-3, which has a role in sperm survival and sperm-oocyte interaction, is also under-expressed in crossbred compared to indigenous spermatozoa. Over-expression of proteins involved in acrosome reaction such as Ataxia telangiectasia and Rad3 related protein (ATR) and inner acrosomal membrane protein (IAM38) (Yu et al., 2006; Maiti et al., 2009) in spermatozoa from crossbred bulls compared to indigenous bulls might be related to premature acrosome reaction in crossbred bull spermatozoa (Aslam, 2014; Aslam et al., 2015). The proteomic analysis of low- and high-fertile spermatozoa revealed that BSP1 and ENO1 as protein biomarkers for low and high fertility in crossbred bulls, respectively (Aslam et al., 2018). The important sperm proteins altered in crossbred bull as compared to indigenous bull spermatozoa are shown in Table 3.

**TABLE 3:** Important sperm proteins downregulated in crossbred bull spermatozoa and indigenous bull spermatozoa (from Aslam, 2014; Aslam et al., 2015).

Protein name	Fold change	Functional significance
Heat shock protein HSP 90-beta	1.7	Cell cycle—Sperm development
60S ribosomal protein L5	3.7	Energy metabolism—Sperm motility
Tubulin beta-3 chain	1.8	Sperm structure—Sperm motility
NADH dehydrogenase	1.8	Energy metabolism—Sperm motility
Prostaglandin E2 receptor EP3	1.8	Fertilization—Sperm capacitation/AR
Radial spoke head protein 9	2.3	Sperm structure—Sperm motility
40S ribosomal protein S29-like	3.5	Energy metabolism—Sperm motility
Beta-defensin 3	2.8	Fertilization—Survival, oocyte interaction
Myosin-13	2.6	Structural integrity—Sperm structure
Myosin-1	2.0	Structural integrity—Sperm structure
WD repeat and FYVE domain-containing protein 1	2.6	Lipid binding—Sperm capacitation
Sperm inner acrosomal membrane protein IAM38	5.1	Fertilization—Zona binding
Zinc finger protein 189/34/789	3.7	Transcription—Sperm formation
Izumo sperm egg fusion protein 4	3.7	Fertilization—Oocyte binding

Although several studies have examined metabolites in human spermatozoa, few studies are available on bull spermatozoa. Recently, metabolites in spermatozoa from high fertility and low fertility crossbred bulls were studied using LC-MS/MS analysis; hypotaurine, L-malic acid, selenocysteine, D-cysteine, and chondroitin 4-sulfate could be markers for crossbred bull fertility (Saraf et al., 2020). Similarly, in a study conducted to assess the sperm metabolomic differences between purebred and crossbred cattle, 1,732 and 1,240 metabolites were identified in purebred and crossbred bull spermatozoa, respectively. Furthermore, aberrations in taurine, hypotaurine and glycerophospholipid metabolism might be associated with the higher incidence of infertility/sub-fertility in crossbred bulls (DasGupta et al., 2021).

In gist, several studies have been conducted on sperm proteomics and transcriptomics in relation to fertility in bull (Moura et al., 2006; Peddinti et al., 2008; D’Amours et al., 2010; Card et al., 2013, 2017), stallion (Suliman et al., 2018; Griffin et al., 2020), and boar (Kwon et al., 2015; Gòdia et al., 2018; Dai et al., 2019; Fraser et al., 2020) and, as a result, few molecular biomarkers for male fertility and semen quality were well established. In case of breeding bulls, proAKAP4 (4MID^®^ technology, SPQI, Lille, France) based kits were developed (Sergeant et al., 2019; Ruelle et al., 2020) for fertility prediction. However, majority of these studies were conducted in purebred bulls. It is pertinent to mention here that breed-to-breed variations were reported in sperm molecular composition; therefore, the available biomolecules and kits for bull fertility prediction need to be tested for its utility in different breeds including crossbred bulls.

### Alterations in Testicular Cells Composition

**FIGURE 3:**
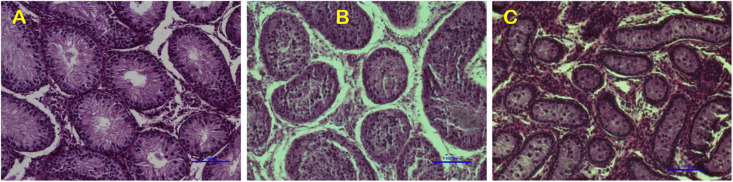
Testicular histology indicating the differences among exotic purebred **(A)**, crossbred **(B)** and indigenous purebred **(C)** males (from Tripathi et al., 2015).

Variations in testicular characteristics between purebred and crossbred bulls are given in Table 4. Earlier studies reported a considerable variation between crossbred and indigenous or exotic purebred cattle in terms of seminiferous tubule morphology and composition. Testicular weight, length, width and volume, as well as scrotal circumference, were higher in crossbred males than zebu males (Brito et al., 2004; Tripathi et al., 2015). The diameter and area of the seminiferous tubules were also greater in crossbred bulls compared to indigenous bulls (Arrighi et al., 2010; Al-Sahaf and Ibrahim, 2012). In another study, both the diameter and area of the seminiferous tubule were higher in Holstein Friesian followed by Holstein Friesian crossbred and zebu males (Tripathi et al., 2015). Besides these anatomical variations, the proportion of Sertoli cells was higher (*p* < 0.05) in purebred bulls compared to crossbred bulls (Tripathi et al., 2015). Comparative histology of seminiferous tubules of Holstein Friesian, Holstein Friesian crossbred, and zebu male is shown in Figure 3. In testicular cytology, the proportion of Sertoli cells ranged between 11 and 14% in purebred males, whereas the proportion of Sertoli cells was only 8–9% in crossbred males. In another study, the true Sertoli cell count was higher in Zebu compared with crossbred males (Rajak et al., 2016a). Yet another interesting finding of this study was that the reduced Sertoli cell count was observed at all age groups of crossbred males (from 1 to 24 months of age). Sertoli cells are known to play a crucial role in spermatogenesis, and alterations in Sertoli cell function may lead to impaired spermatogenesis and male infertility. The daily sperm production and testicular size in adult testis depends upon the number of Sertoli cells (Sharpe et al., 2003). Furthermore, Sertoli cells provide critical factors for germ cell development, either in physical support or biochemical stimulation in the form of nutrients and growth factors (Russell and Griswold, 1993). A recent study by Rajak et al. (2016b) showed that “good” bulls had a significantly higher (25%) proportion of Sertoli cells in testicular cytology compared to “poor” bulls (11%). Sertoli cells are capable of supporting only a finite number of germ cells; the final number of Sertoli cells in a given male sets the upper limit for testicular sperm production (Waqas et al., 2019) and determines the level of male fertility (Yan et al., 2020). A high number of Sertoli cells per spermatogenic cell would be expected to provide sufficient support and nourishment for successful progression of spermatogenesis and for production of good-quality spermatozoa. However, existing reports clearly indicate that crossbred bull testis contain a lower number of Sertoli cells than purebred bulls. Therefore, the poor semen quality and high incidence of sub-fertility in crossbred males might be due to the reduced number of Sertoli cells in their testis.

**TABLE 4:** Testicular characteristics between purebred and crossbred bulls (based on the data from Brito et al., 2004; Tripathi et al., 2015).

Testicular characteristics	Purebred bulls	Crossbred bulls
Scrotal circumference (cm)	27.2 ± 1.9 (Nellore)	31.2 ± 2.2 (Charolais × Zebu)
Testes volume (cm^3^)	148.3 ± 33.1 (Nellore)	242.5 ± 51.4 (Charolais × Zebu)
Testicular artery length (cm)	147.2 ± 27.3 (Nellore)	222.1 ± 42.1 (Charolais × Zebu)
Testicular artery volume (ml)	5.0 ± 1.7 (Nellore)	11.4 ± 4.4 (Charolais × Zebu)
Sertoli cell %	14.66 (Tharparkar)	8.5 (HF × Tharparkar)
Diameter of the seminiferous tubule (Mean ± SE)	111.71 ± 2.53 (Tharparkar)	183.43 ± 4.57 (HF × Tharparkar)
Daily sperm production per gram of testicular parenchyma (× 10^6^)	10.8 ± 2.8 (Nellore)	10.3 ± 2.8 (Charolais × Zebu)
Epididymal sperm reserve (× 10^9^)	7.0 ± 3.5 (Nellore)	18.2 ± 9.6 (Charolais × Zebu)

### Alterations in Testicular Molecular Health

**FIGURE 4:**
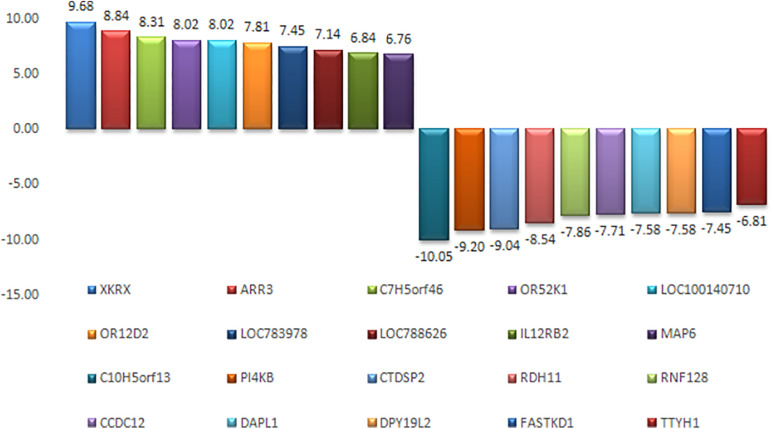
Top 10 upregulated and top 10 downregulated transcripts in crossbred bull testes as compared to indigenous bull testes (from Elango et al., 2020).

**FIGURE 5:**
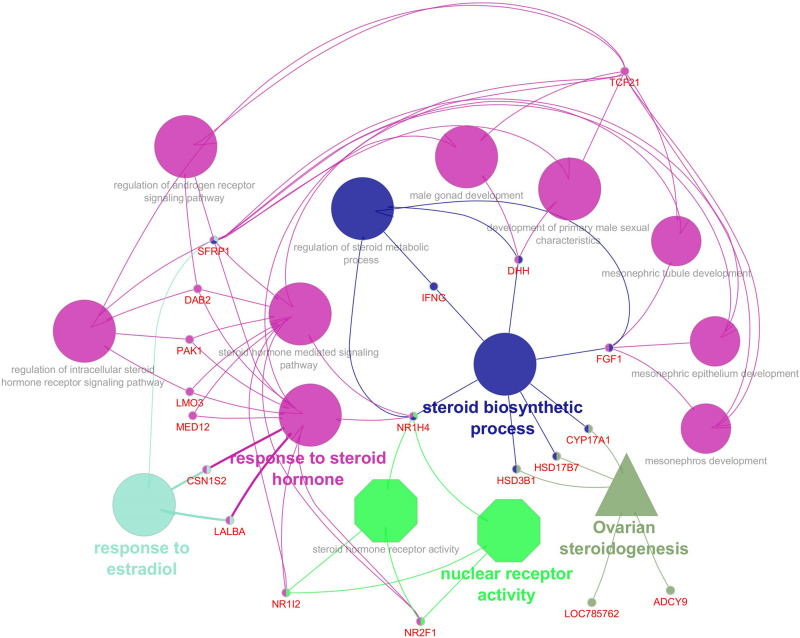
Downregulated steroidogenesis related genes in crossbred testis and their biological process (Elliptical), molecular functions (octagonal) and pathway (triangle) (from Elango et al., 2020).

An appropriate molecular environment in the testis is required for proper spermatogenesis. In a recent study, the testicular transcripts related to sperm function and fertilizing potential were altered in crossbred bull testis compared to purebred bull testis (Elango et al., 2020). This study identified a total of 1,466 transcripts that were differentially expressed between crossbred and indigenous bull testes. Among these, 1,038 transcripts were upregulated, and 428 transcripts were downregulated in crossbred bulls compared to indigenous bull testes; the top 10 upregulated and downregulated transcripts are shown in Figure 4. Furthermore, the *DPY19L2* and *PI4KB* genes, reported to be involved in sperm acrosome formation and capacitation, respectively, were significantly downregulated in crossbred testis. Moreover, genes involved in proteolysis and ubiquitination (a final stage of apoptosis in testis) were upregulated, whereas genes involved in WNT pathway (involved in sperm motility initiation and inhibition of ubiquitination) were downregulated in crossbred testes. The genes involved in steroidogenesis, including *CYP17A1* gene (involved in 17, 20-lyase activity and 17 α-hydroxylase activity, which are vital for steroidogenesis) were downregulated in crossbred bull testis. Genes associated with steroidogenesis are downregulated in crossbred bull testis compared to purebred testis. Downregulated steroidogenesis-related genes and their involvement as a group in different biological process, molecular function and pathway are shown in Figure 5. Besides this, the downregulation of GABAergic synapse pathway (vital for progesterone mediated sperm function) also collectively indicated the problems in steroidogenesis in crossbred bulls. The genes involved in cell proliferation, differentiation and cell population maintenance were also downregulated in crossbred bull testis. Thus, downregulation of genes associated with spermatogenesis and steroidogenesis in crossbred bulls could be the reason for infertility compared to purebred bulls.

In a study by Tripathi et al. (2014), 219 protein spots and 15 protein spots were under- expressed and 7 protein spots were over-expressed in spermatogenic cells of indigenous bulls compared to crossbred bulls. The protein PEBP is highly expressed in spermatogenic cells of crossbred bulls. It is restricted to spermatids and involved in membrane organization in spermatozoa. RINGO/Speedy-A is over-expressed in the Sertoli cells of crossbred bulls. Over-expression of this protein may indicate the accumulation of high levels of the speedy-A protein that will interfere with chromatin de-condensation (Tripathi et al., 2014). In another study that compared the proteomic profile of spermatogenic cells derived from crossbred and purebred bulls, 79 proteins were differentially expressed (Tomar et al., 2021). The proteins associated with sperm function and fertilization processes, such as calumenin, prosaposin, vimentin, GRP78, and APOA1, were downregulated in crossbred bulls, which might be associated with the high incidence of sub-fertility in these bulls. These studies indicate that the testicular environment is altered in crossbred bulls compared to purebred bulls.

### Reproductive Endocrinological Differences

**FIGURE 6:**
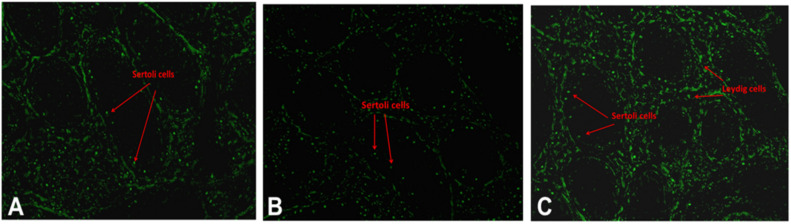
Immunolocalization of Sertoli and Leydig cells using GATA4 in testis of exotic purebred **(A)**, crossbred **(B)** and indigenous purebred **(C)** males (from Tripathi et al., 2015).

Successful reproduction depends upon an optimal endocrine milieu. Development of testis, puberty, sexual maturity and spermatogenesis require certain levels of reproductive hormones, mainly follicle stimulating hormone (FSH), luteinizing hormone (LH), and testosterone (O’Donnell et al., 2006). Although wide variations in the circulating concentrations of FSH, LH and testosterone have been detected among different bovine breeds (Moura et al., 2011), the reports were not consistent. High serum testosterone concentrations were associated with poor semen quality in crossbred bulls (Sharma et al., 1987), whereas Gulia et al. (2010) and Rajak et al. (2016a) observed that testosterone concentrations were significantly lower in crossbred bulls compared to indigenous bulls. The testosterone produced from the interstitial cells (Leydig cells) under the influence of LH hormone, is important for normal spermatogenesis and male characteristics. Furthermore, the increase in testosterone concentrations in relation to age was very rapid in indigenous bulls, whereas in crossbred bulls the increase was very low and linear (Gulia et al., 2010). Similarly, higher LH and testosterone concentrations were noticed at 18 months of age in crossbred bulls, compared to 24 months in indigenous bulls, which might explain early sexual maturity in crossbred males compared to zebu males (Rajak et al., 2016a). Anti-Mullerian hormone (AMH), secreted by the Sertoli cells, plays a major role during sexual differentiation and regression of Mullerian duct during early life, but is also detected in adult males (Josso et al., 2001). In human beings, serum AMH concentrations seem to constitute additional diagnostic parameters for male subfertility as they reflect Sertoli cell function (Iliadou et al., 2015). Little information is available on the relationship between circulatory AMH concentrations and bull fertility. In a preliminary study on this aspect, a lower concentration of AMH was observed in crossbred bulls than in purebred adult bulls (Rajak et al., 2016a). In this study, the transcriptional abundance on the AMH gene was higher in purebred than crossbred males. These observations on reduced serum AMH concentrations in crossbred bulls also support earlier finding that the number of Sertoli cells are lower in crossbred bulls compared to purebred bulls (Tripathi et al., 2015). In addition, Sertoli cells produce inhibin, activin (involved in maintaining FSH secretion) and androgen binding protein (maintain testosterone concentration in seminiferous tubules); therefore, reduced Sertoli cell number in crossbred bulls may alter FSH secretion and testosterone concentration. Collectively, all these findings indicate that Sertoli cell counts are an important factor associated with fertility or sub-fertility in crossbred bulls. The immunolocalization of Sertoli cells and Leydig cells indicating the differences between crossbred and purebred bull testis is shown in Figure 6. In addition to altered Sertoli and Leydig cell composition, the altered steroidogenesis-related transcripts after crossbreeding due to the genetic incompatibility between the parent breeds (Elango et al., 2020) is also a major reason for the endocrinological difference in crossbred bulls. It was described in detail in the previous section.

### Gene and Chromosomal Alterations

**FIGURE 7:**
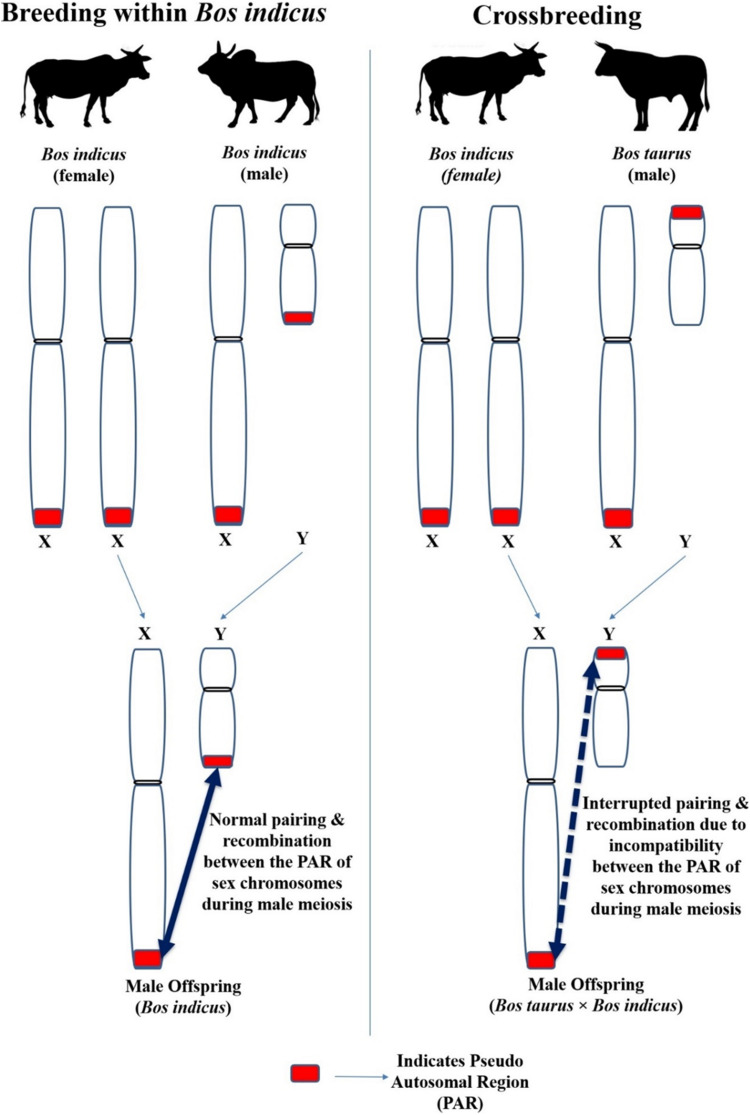
The impact of different location of PAR between the Y chromosome of *Bos taurus* and *Bos indicus* on the X-Y pairing and recombination (crossing over) during male meiosis in crossbred bulls.

Although Y chromosomes are commonly small, their size, shape and genetic makeup differ between different species (Di Meo et al., 2005). Moreover, the length of Y chromosome differed significantly between different breeds of cattle (Halnan and Watson, 1982) including crossbred (*Bos taurus* × *Bos indicus*) cattle (Mandal and Sharma, 2003). The X and Y chromosome contain the short segment of identical nucleotide sequence (98–100%) located in the terminal portion of their respective short or long arms; this sequence of homology is known as the pseudo autosomal region (PAR; indicated as red color box in Figure 7). This PAR possesses different functional and molecular characteristics than the autosomes and the remaining areas of the sex chromosomes (Das et al., 2009). Synapse and recombination (crossing over) occurring between the PAR of X and Y chromosome during the prophase of male meiosis is indispensable for the normal separation of the sex chromosome into different spermatids. Meiosis will not be completed in cells in which X-Y recombination has not occurred (Kauppi et al., 2011; Raudsepp and Chowdhary, 2015).

Inter-species breeding can cause structural and/or molecular/genetic discrepancy between the PAR of X and Y chromosome in the offspring and interferes in the usual synapse and recombination during male meiosis, resulting in infertility (Blaschke and Rappold, 2006). For instance, the Y chromosome of cattle-yak F1 hybrid and yak is submetacentric but is metacentric in Chinese Yellow cattle (Bos taurus). The Y chromosome length in cattle-yak is longer (Guo, 1983) than that observed in their parents (Chinese Yellow cattle and Yak). The variation between Y chromosome size and morphology between the parents causes inherent imbalance between the X and Y chromosomes, and in their respective PAR in the offspring (Zhang et al., 2016).

The same reasoning can be speculated for the progeny born out of the crossbreeding *Bos taurus* with *Bos indicus*, because the Y chromosome of *Bos taurus* is submetacentric whereas in *Bos indicus* it is acrocentric with visible p-arms. The Y chromosomal arms are of different sizes in *Bos indicus* and *Bos taurus*. Furthermore, the location of PAR on the Y chromosome differs as it is located on the short arm of *Bos taurus* Y chromosome but on the long arm of *Bos indicus* Y chromosome. The difference between the Y chromosome of *Bos taurus* × *Bos indicus* is due to pericentric inversion or centromere transposition. The Crossbred (*Bos taurus* × *Bos indicus*) bulls have a submetacentric Y chromosome (Mandal and Sharma, 2003; Di Meo et al., 2005; Raudsepp et al., 2012; Mukherjee et al., 2015). As mentioned above for species hybridization between cattle and yak, crossbreeding between *Bos taurus* and *Bos indicus* (which have different Y chromosome morphology and a different location of PAR in the Y chromosome) can result in structural and/or molecular differences between the PAR of X and Y chromosome in the male offspring. Thus, normal pairing and recombination between the PAR of X and Y chromosome during male meiosis would be disrupted. This may eventually lead to improper spermatogenesis and infertility in crossbred bulls. Although the location of PAR on the short or long arm does not completely block the pairing between X and Y chromosomes, it interrupts the success of X-Y pairing by having genetic consequences in structural chromosomal reorganization. For instance, isochromosome formation results in duplication or complete deletion in one of the sex chromosomal regions (Raudsepp et al., 2012).

Disparity between sex chromosomes due to species hybridization or crossbreeding may also lead to deletions and duplication of genes in the offspring. This is supported by the findings of Mukherjee et al. (2013), who reported copy number variations in the Y chromosomal genes [Sex-determining gene on Y chromosome (*SRY*), DEAD box polypeptide 3-Y chromosome (*DDX3Y*) and Testis-specific protein on Y chromosome (*TSPY*)] of Holstein Friesian crossbred bulls compared to Sahiwal (*Bos indicus*) bulls. Furthermore, Zhang et al. (2016) also reported enormous copy number variations in the Y chromosomal genes, such as *PRAMEY* (Preferentially expressed antigen in melanoma, Y-linked), *TSPY* (Testis-specific protein, Y-encoded), *ZNF280BY* (Zinc finger protein 280B, Y-linked), and *HSFY* (Heat-shock transcription factor, Y-linked) in the Y chromosome of F1 hybrid cattle-yak bulls than in cattle bulls. However, in-depth studies involving a large number of different purebred and crossbred males are required to understand the effect of a discrepancy between the PAR of X and Y chromosome on male fertility.

## Conclusion and Future Perspectives

**FIGURE 8:**
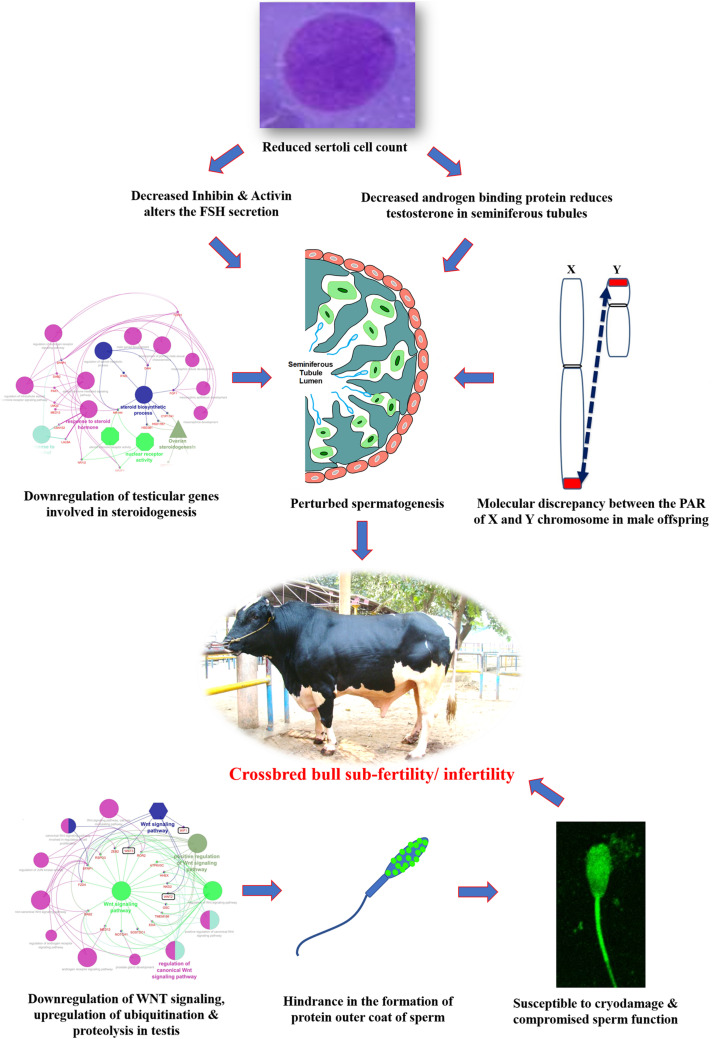
Hypothetical model indicating the possible etiologies of crossbred bull subfertility/infertility.

Undoubtfully, crossbreeding of low-producing zebu cows with exotic bulls of high genetic merit has resulted in the production of superior genotypes with hybrid vigor and enhanced milk production efficiency. On the other hand, it is evident from the foregoing information that the magnitude of reproductive problems is higher in crossbred bulls compared to purebred bulls. Until recently, little research had been done to identify the underlying etiological factors for this high incidence of infertility/sub-fertility in crossbred bulls. During the last decade, considerable research has been conducted; published information suggests alterations at the level of testis, spermatozoa, seminal plasma and male reproductive hormones, in crossbred bulls compared to purebred bulls. It is also evident that crossbreeding of *Bos taurus* with *Bos indicus* might eventually lead to improper spermatogenesis and infertility in crossbred bulls because of differences in the Y chromosome. The Y chromosome of *Bos taurus* is submetacentric, whereas in *Bos indicus* it is acrocentric. Differences in location of PAR in the Y chromosome might disrupt the process of normal pairing and recombination between the PAR of X and Y chromosome during male meiosis. Information about the influence of the level of exotic blood on fertility in crossbred males is very limited. Schematic representation of the possible cellular and molecular alterations in crossbred testis and spermatozoa that might culminate in subfertility/infertility is given in Figure 8.

Detailed and large-scale studies involving crossbred males with different levels of exotic inheritance are required to have a clear understanding about infertility/sub-fertility in crossbred bulls. Genes in the pseudo autosomal regions of X and Y chromosome of *Bos taurus*, *Bos indicus*, and their crossbred male offspring should be studied to understand the origin of these reproductive problems in crossbred males. Expression of genes such as *DDX3Y*, *RINGO*, and *SPATA7* and metabolites such as hypotaurine, L-malic acid, selenocysteine, D-cysteine, and chondroitin 4-sulfate are to be studied further to determine their role in crossbred male reproduction. Testicular transcriptomic study indicated an inability of the crossbred testis to maintain protein stability and steroidogenesis, which could be the pressure point for reproductive problems in crossbred bulls. Therefore, research to understand and improve protein stability and steroidogenesis in crossbred bulls will be important in the future. In addition, tailored freezing techniques and specific extenders need to be tested because poor freezability of spermatozoa is an important problem in crossbred bulls. Although sperm mRNAs (messenger RNA) were studied to some extent in crossbred bulls, the other RNAs such as transfer RNA (tRNA), ribosomal RNA (rRNA), long non-coding RNA (lncRNA), mitochondrial RNA (mt-RNA), small non-coding RNA (sncRNA), small nuclear RNA (snRNA), and small nucleolar RNA (snoRNA) need to be investigated to broaden our understanding about crossbred bull fertility. Studies involving microRNA, small interfering RNA, their intracellular delivery and target genes in crossbred bulls are particularly warranted. Furthermore, use of technologies such as gene transfer, editing, slicing and knockout may be useful in future to understand and improve the fertility in crossbred bulls.

## Author Contributions

AK, KE, and TD conceptualized the review. All authors were involved in literature review and development of the manuscript, and approved the manuscript for publication.

## Conflict of Interest

The authors declare that the research was conducted in the absence of any commercial or financial relationships that could be construed as a potential conflict of interest.
